# Despite plasticity, heatwaves are costly for a coral reef fish

**DOI:** 10.1038/s41598-024-63273-8

**Published:** 2024-06-10

**Authors:** Jacey C. Van Wert, Kim Birnie-Gauvin, Jordan Gallagher, Emily A. Hardison, Kaitlyn Landfield, Deron E. Burkepile, Erika J. Eliason

**Affiliations:** 1 Department of Ecology, Evolution & Marine Biology, University of California, Santa Barbara, CA 93106 USA; 2 Section for Freshwater Fisheries and Ecology, National Institute of Aquatic Resources, Technical University of Denmark, Silkeborg, Denmark; 3 Marine Science Institute, University of California, Santa Barbara, Santa Barbara, CA 93106 USA

**Keywords:** Climate change, Ecophysiology, Heart rate, Metabolism, Thermal tolerance, Climate-change ecology, Ecophysiology

## Abstract

Climate change is intensifying extreme weather events, including marine heatwaves, which are prolonged periods of anomalously high sea surface temperature that pose a novel threat to aquatic animals. Tropical animals may be especially vulnerable to marine heatwaves because they are adapted to a narrow temperature range. If these animals cannot acclimate to marine heatwaves, the extreme heat could impair their behavior and fitness. Here, we investigated how marine heatwave conditions affected the performance and thermal tolerance of a tropical predatory fish, arceye hawkfish (*Paracirrhites arcatu*s), across two seasons in Moorea, French Polynesia. We found that the fish’s daily activities, including recovery from burst swimming and digestion, were more energetically costly in fish exposed to marine heatwave conditions across both seasons, while their aerobic capacity remained the same. Given their constrained energy budget, these rising costs associated with warming may impact how hawkfish prioritize activities. Additionally, hawkfish that were exposed to hotter temperatures exhibited cardiac plasticity by increasing their maximum heart rate but were still operating within a few degrees of their thermal limits. With more frequent and intense heatwaves, hawkfish, and other tropical fishes must rapidly acclimate, or they may suffer physiological consequences that alter their role in the ecosystem.

## Introduction

As global biodiversity faces more frequent and intense stressors associated with climate change, species must acclimate, adapt, move, or die^1^. Given that adaptation occurs over generations and relocation requires both dispersal capacity and available habitat conditions, acclimation may be the only coping mechanism available to many species^2^. In contrast to long-term ocean warming, marine heatwaves ensue rapidly, with anomalous sea surface temperatures surpassing average temperatures by approximately 2–4 °C for ≥ 5 days to multiple weeks^3^. Thus, an animal’s capacity to acclimate to these conditions is fundamental to its survival, and these challenges are likely to be particularly acute for species in tropical ecosystems such as coral reefs that exist near their thermal maxima^4^.

Coral reef fish evolved in the stable thermal environment of the tropics and perform optimally within narrow temperature ranges, living close to their thermal limits^5^. Fish physiology may be sensitive to temperature changes at the seasonal scale^6,7^. This begets the question of how these fish cope with marine heatwaves during seasonal extremes. Performance across levels of biological organization can be compromised at extreme temperatures^5,8^. Aerobic scope (i.e., the difference between standard metabolism and maximal metabolism) reflects the capacity of the fish to perform essential behaviors, including swimming, digestion, defense, and reproduction^9^. Reef fishes are critical for maintaining coral reefs by providing ecological services such as recycling nutrients via egestion and excretion, guarding territories, and clearing space^10^, and all these activities require sufficient aerobic capacity. Previous work shows that small increases in temperature can have sublethal effects on fish, including reducing their aerobic scope^11–15^.

At the same time, other vital processes may become more energetically costly at high temperatures, such as digestion (specific dynamic action, SDA)^16^ and recovery from exertion^17^. Thus, during marine heatwaves, fish may be less able to perform certain activities (i.e., swimming, eating, reproduction, or territoriality) within their constrained energy budget^18,19^. Furthermore, if digestive costs increase during marine heatwaves, fish may need to reduce their consumption rate to maintain scope^20^. Fish presence increases coral performance during heat stress by excreting nutrients that enhance coral growth and coral nutritional reserves^21–23^, making fish’s ability to survive and perform during heatwaves especially critical. If fish cannot fulfill their ecological roles because of constrained physiological performance during a marine heatwave, reef ecosystems may be threatened^24^.

The function of the heart may be particularly vulnerable to marine heatwaves, compromising its key role in transporting oxygen, nutrients, wastes, hormones, and immune cells. As whole animal oxygen demand increases with warming, the heart ensures sufficient oxygen delivery to the tissues primarily via an increase in heart rate^25–27^. At some critical temperature however, maximum heart rate (*f*_Hmax_) can no longer increase and eventually becomes arrhythmic^28^. Heart failure, therefore, is considered a primary mechanism that regulates the upper thermal limits of fish^25^. In warm-temperate systems, marine heatwaves have already exceeded the cardiac thermal limits in a sparid fish and may compromise its survival and distribution^29^. Cardiac thermal limits have yet to be elucidated in coral reef fish. Accordingly, measuring the thermal limits of the heart in coral reef fish may help us assess the vulnerability of reef fish to climate change.

**Figure 1:**
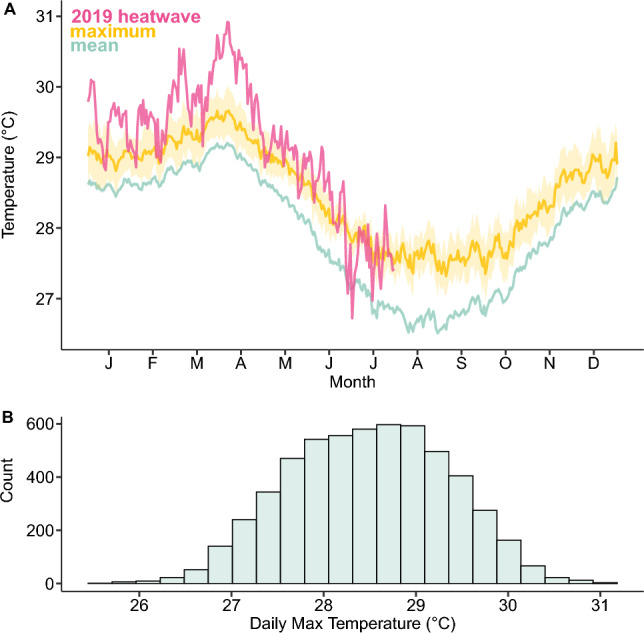
Temperatures (°C) on Moorea backreef between 2005 and 2021. (**A**) The daily mean (turquoise) and maximum (yellow) temperatures averaged across 2005–2021, and daily maximum (pink) temperatures during a marine heatwave in 2019. The 95% confidence interval is shaded and displayed for the daily mean (turquoise) and maximum (yellow); (**B**) overall count in daily maximum temperatures over the 16-year timeframe. Temperature data is from LTER2 site^31^, 500 m from the hawkfish collection site.

In this study, our overarching goal was to determine how marine heatwaves impact the performance of a common coral reef fish. The arceye hawkfish (*Paracirrhites arcatus*) is a “perching” ambush predator that depends on its ability to briefly perform at high exertion levels to capture prey, making this species an excellent model organism for assessing digestive, recovery, and cardiac performance under heatwave conditions^30^. The work took place in Moorea, French Polynesia, where daily maximum ocean temperatures typically ranged from 26 to 29 °C annually but reached temperatures beyond 29 °C during marine heatwave events (Fig. 1). Our first objective was to (1) determine if various whole-organism physiological performance traits are impaired after a week-long heatwave event during seasonal extremes. We assessed the metabolic rates, aerobic scope, exercise recovery, and SDA of fish acclimated to Austral winter (27, 31 °C) and Austral summer (28, 29, 33 °C) marine heatwave conditions under current and Intergovernmental Panel on Climate Change (IPCC) projections (i.e., + 1, + 4, or + 5 °C). Our second objective was to (2) determine if relative meal size (2 vs. 4% body mass) increases costs for fish acclimated to higher temperatures. Our final goal was to (3) assess whether the cardiac performance and cardiac upper thermal limits are impaired in hawkfish under marine heatwave scenarios. Collectively, these metrics provide insight into how hawkfish may be able to perform essential activities, including hunting and maintaining their dominance over coral heads during a marine heatwave.

## Results

### Oxygen consumption rates

**Figure 2:**
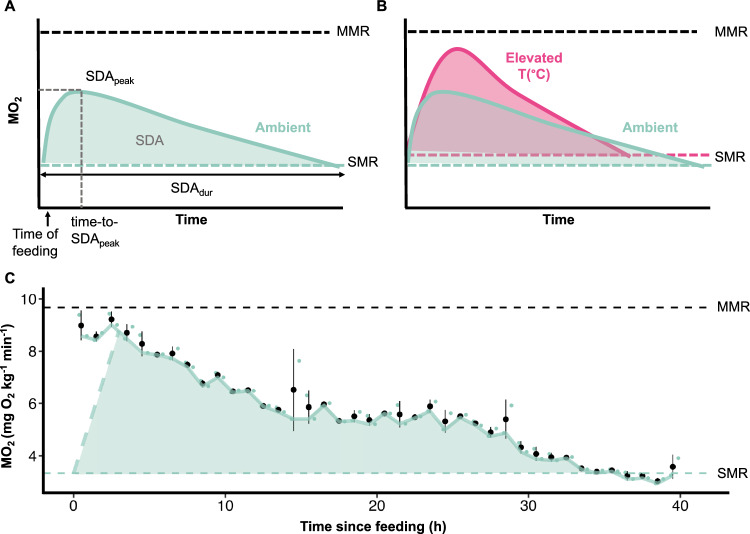
Conceptual and representative diagrams of SDA after a single feeding event at hour 0. (**A**) The line represents the MO_2_ values post-feeding. SDA is the integral under the curve between postprandial MO_2_ and standard metabolic rate (SMR, turquoise dashed line) over SDA_dur_ (the duration between feeding and the first value to fall below SMR). Peak SDA (SDA_peak_) is the maximal postprandial MO_2_ value (not pooled) following feeding and time-to-SDA_peak_ is the associated time (h) until peak SDA. (**B**) Expected MO_2_ trace for fish under elevated temperatures in comparison to ambient temperatures. (**C**) A representative trace of MO_2_ for an ambient fish with each black point as mean MO_2_ ± SEM pooled for every hour and each turquoise point as an individual measurement. The turquoise dashed line indicates where the SDA calculations begin to control for the effect of handling (anesthetic and gavage), and the horizontal lines indicate the MMR and SMR values for this individual fish. The turquoise solid line connects the lowest MO_2_ of each hour. The SDA is calculated as the shaded area between the dashed turquoise line, the solid turquoise line, and SMR until SDA ends.

**Figure 3:**
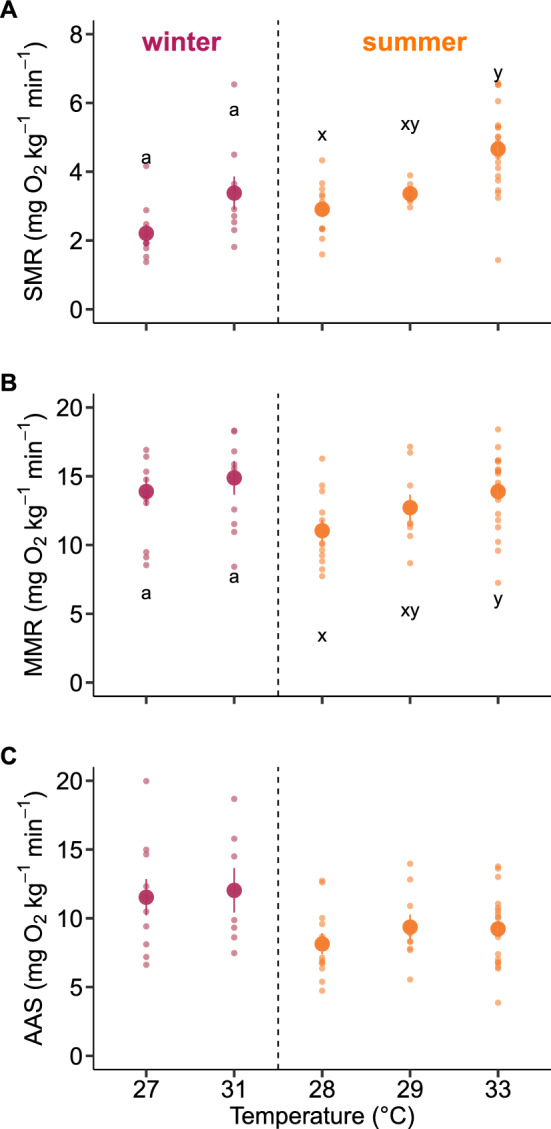
Oxygen uptake rate (mg O_2_ kg^−1^ min^−1^) in hawkfish acclimated during the winter (pink; 27 and 31 °C) and summer (orange; 28, 29 and 33 °C). (**A**) Standard Metabolic Rate (SMR, mg O_2_ kg^−1^ min^−1^). (**B**) Maximum Metabolic Rate (MMR, mg O_2_ kg^−1^ min^−1^). (**C**) Absolute Aerobic Scope (AAS, mg O_2_ kg^−1^ min^−1^). Large data points and error bars represent mean ± SEM, and data from individuals are plotted as small data points. Statistics are assessed for winter (pink) and summer (orange) separately. Lowercase letters indicate significant differences (P < 0.05) between acclimation temperatures within a season (ANOVA). Note that 27 and 28 °C represent ambient temperatures in the wild for the winter and summer, respectively, thus both acting as controls for the respective seasons.

We assessed the whole-organism physiological performance of hawkfish under heatwave conditions via various metabolic performance traits (Fig. 2). We found that SMR generally increased with acclimation temperature, though the only significant increase occurred in fish acclimated to 33 °C, which was 60% greater than fish acclimated to 28 °C (Fig. 3A, Supplementary Table S2). In contrast, MMR was consistent across acclimation temperatures in the winter but increased in the summer by 27% from ambient to 33 °C (P = 0.026, ANOVA). AAS did not vary across acclimation temperatures in the summer (P = 0.461, ANOVA) or winter (P = 0.809, ANOVA) (Fig. 3B,C, Supplementary Table S2).

**Figure 4:**
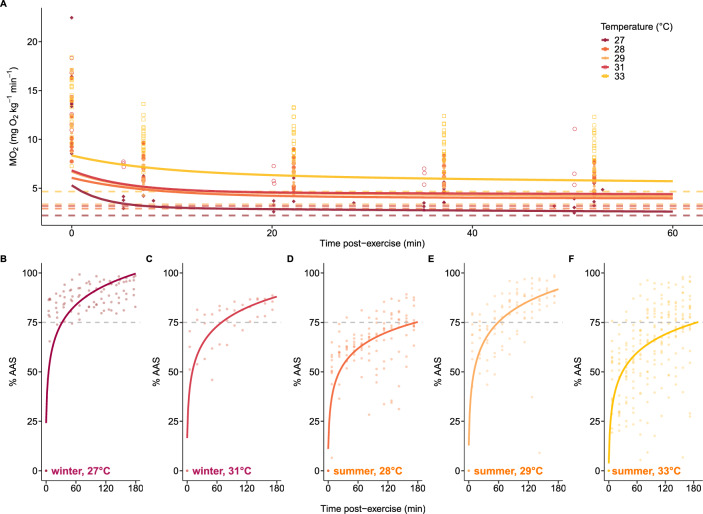
Recovery after exhaustive exercise for fish acclimated during the winter (27 and 31 °C) and summer (28, 29 and 33 °C). (**A**) Metabolic rate (MO_2_) post chase during the first 60 min of recovery. Biexponential curves are fit for each acclimation temperature and each data point represents a measurement at a timepoint for an individual fish. Dashed lines represent average SMR for that acclimation temperature. (**B–F**) Percent absolute aerobic scope (%AAS) recovered during the first 180 min following exhaustive exercise for fish acclimated in the winter (pinks): 27 [N = 8] and 31 °C [N = 4] and summer (oranges): 28 [N = 10], 29 [N = 9], and 33 °C [N = 19]). Values at 0 indicate MMR. Logarithmic growth curves are fit to each acclimation temperature as described in the methods. The grey horizontal line marks the point at which each treatment reaches 75% AAS. Individual data points are presented for each fish at each timepoint.

Another whole-organism physiological metric we examined was post-exercise recovery, which differed across acclimation temperatures. Fish acclimated to 27 °C had a rapid recovery (sharp decline in MO_2_), whereas fish acclimated to 33 °C had a slower recovery (slow decline in MO_2_) (Fig. 4A). FAS available to fish surpassed 2 in 10 min for 27 °C fish, 100 min for 28 °C fish, 55 min for 29 °C fish, 10 min for 31 °C fish and 145 min for 33 °C fish. Fish recovered to 75% of AAS at 33 min for 27 °C fish, 180 min for 28 °C fish, 60 min for 29 °C fish, 69 min for 31 °C fish, and 188 min for 33 °C fish (Fig. 4B–F). Meanwhile, only 27 °C acclimated fish recovered to 100% AAS within 180 min after the exhaustive exercise (Fig. 4B–F). The variability in %AAS recovered generally increased with temperature (27 °C: 16.7% (coefficient of variation); 28 °C: 19.1%, 29 °C: 19.4%, 31 °C: 18.1%, 33 °C: 21.6%) (Fig. 4B–F).

**Figure 5:**
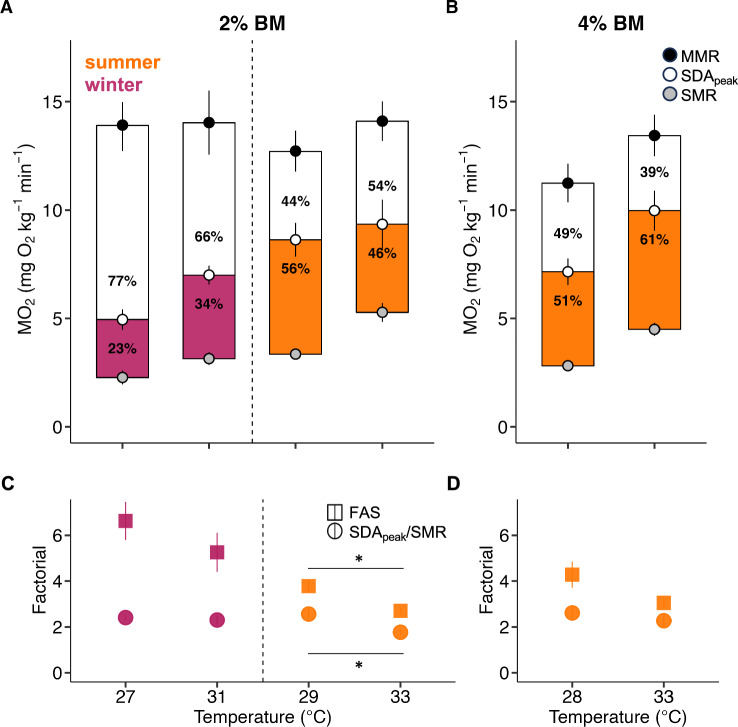
Scope for activity during SDA. Data shown for acclimation temperatures fed 2% body mass (BM) (27 [N = 10], 29 [N = 9], 31 [N = 7], and 33 °C [N = 7]) (first panel) or 28 °C and 33 °C 4% BM (28 [N = 10], and 33 °C [N = 11]) (second panel). (**A,B**) Mean metabolic rates are shown for hawkfish at rest (SMR; gray), at maximal activity (MMR; black), and at maximal digestion (SDA_peak_; white) with SEM as the error. The colored bars (pink and orange) and associated percentages indicate the mean percent of scope used for SDA, and the remaining area in white and its associated percentage is the mean scope for activity. These values are not statistically compared and are reported for illustrative purposes. (**C,D**) Factorial of MMR/SMR (FAS; square) and SDA_peak_/SMR (circle). The difference between FAS and SDA_peak_/SMR indicates the extent to which fish experience a metabolic constraint. Statistics are assessed for winter (pink) and summer (orange) separately. Asterisks indicate significant differences (P < 0.05) between acclimation temperatures within a season (t-test).

The final metric we examined to assess whole-organism physiological performance was SDA when fed 2% BM (Table 1). When comparing SDA across acclimation temperatures, we found an effect of temperature on SDA_peak_ in the winter. SDA_peak_ increased by nearly 50% with increasing acclimation temperature, from 4.95 mg O_2_ kg^−1^ min^−1^ at 27 °C to 7.26 mg O_2_ kg^−1^ min^−1^ at 31 °C (P = 0.003; Table 1, Supplementary Table S3). There was no effect of temperature on SDA or SDA_dur_ (Supplementary Table S3). Overall, the average remaining scope for activity available to fish during SDA_peak_ during the digestion of a 2% BM meal decreased by 47%, from 8.95 to 4.75 mg O_2_ kg^−1^ min^−1^ (Fig. 5A). FAS was greatest in the winter at 27 and 31 °C and decreased in the summer at 29, and 33 °C (Fig. 5C). Thus, while MO_2_ could be increased beyond SMR by nearly sixfold at 27 and 31 °C, the fish in the summer acclimated to 29 °C could only increase MO_2_ above SMR by fourfold and the 33 °C fish by threefold (P < 0.01, t-test) (Fig. 5C). SDA_peak_ as a ratio of SMR remained close to 2 across all acclimation temperatures and was highest at 29 °C in the summer.

When we tested if relative meal size (2 vs 4% body mass) would increase costs for fish acclimated to 33 °C, we did not find a strong response. SDA was not greater in fish fed 4% BM (52.56 mg O_2_ kg^−1^) compared to their counterpart fed 2% BM (36.62 mg O_2_ kg^−1^) at 33 °C (P = 0.292; Fig. 5A,B; Supplementary Table S4). Also surprisingly, relative meal size did not impact SDA_peak_ (P = 0.660; Supplementary Table S4). Overall, there was an effect of acclimation temperature, with the highest SDA_peak_ at 33 °C reaching 9.98 mg O_2_ kg^−1^ min^−1^ in fish fed 4% BM (Table 1). Meanwhile, fish fed 4% BM acclimated to ambient temperature had a marginally greater FAS than those acclimated to 33 °C (P = 0.053) and SDA_peak_/SMR also remained close to 2 (Fig. 5D).

**Table 1:** Summary statistics for SDA metabolism.

Acclimation temperature (°C)	BM fed (%)	n	SDA (mg O_2_ kg^−1^)	SDA_dur_ (h)	SDA_peak_ (mg O_2_ kg^−1^ min^−1^)	time-to-SDA_peak_ (h)	SDA_coeff_ (%)
27	2	10	31.23 ± 4.24	28.1 ± 1.42	4.95 ± 0.47	13.6 ± 2.94	0.69 ± 0.09
28	4	10	57.44 ± 8.43	33.28 ± 1.01	7.16 ± 0.61	6.15 ± 1.6	1.26 ± 0.19
29	2	9	48.58 ± 6.96	31.94 ± 1.44	8.63 ± 0.78	6.61 ± 2.9	1.07 ± 0.15
31	2	8	30.3 ± 4.78	24.12 ± 2.38	7.26 ± 0.46	7.94 ± 1.73	0.67 ± 0.11
33	2	7	36.62 ± 12.77	25.83 ± 6.99	9.35 ± 1.13	3.5 ± 0.94	0.8 ± 0.28
	4	11	52.56 ± 6.28	25.94 ± 1.46	9.98 ± 0.92	5.23 ± 1.52	1.15 ± 0.14

SDA metrics (sample size (n), SDA, SDA_dur_, SDA_peak_, time-to-SDA_peak,_ and SDA_coeff_) across 5 acclimation temperatures and 2 feeding treatments (2% and 4% body mass (BM) of scallop). See Supplementary Tables S3 and S4 for statistical results.

### Cardiac performance

**Figure 6:**
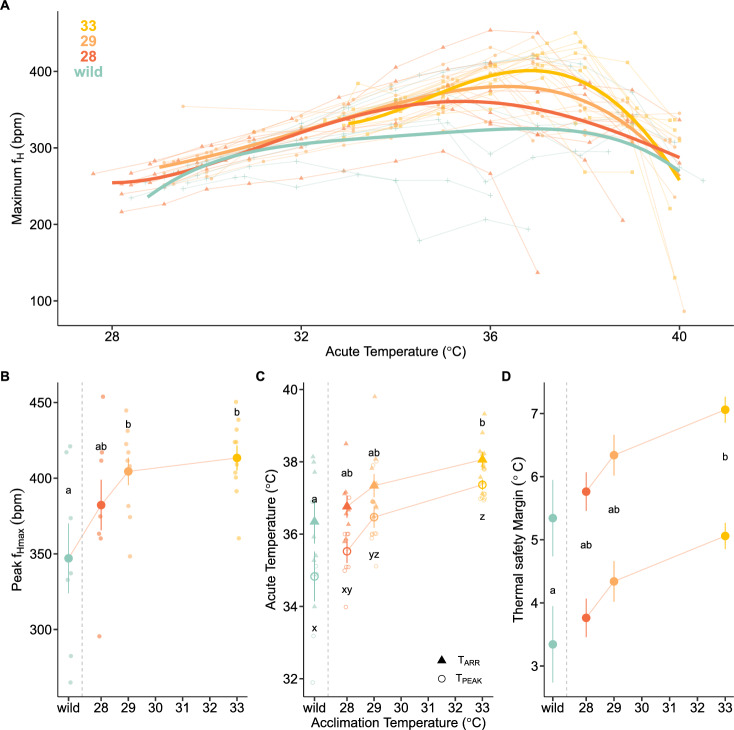
Acute cardiac thermal performance curve and associated metrics of wild-caught (28 °C, turquoise), and lab-acclimated hawkfish: 28 °C (red–orange), 29 °C (orange), and 33 °C (yellow). (**A**) Individual performance curves and data points overlaid with thermal performance curves modeled as a fourth-order polynomial across each acclimation temperature as determined by the best-fit model by BIC (Supplementary Table S5). (**B**) Peak maximum heart rate (*f*_Hmax_) in bpm. (**C**) temperature at *f*_Hmax_ (T_PEAK_; circles, significance letters below) and temperature at which arrhythmias began (T_ARR_; triangles, significance letters above), and (**D**) thermal safety margin presented as the difference between T_ARR_ and maximum environmental temperature during a marine heatwave of 31 °C (top) or 33 °C (bottom). (**B–D**) Values are presented as means ± SEM and individual points represent values for individuals. Different lowercase letters indicate significant differences among acclimation temperatures (ANOVA, Tukey HSD). The gray dashed vertical line separates wild-caught fish from lab-acclimated fish.

We assessed the cardiac performance and upper thermal limits under heatwave scenarios using the cardiac thermal tolerance test. For each acclimation temperature, *f*_Hmax_ followed the expected shape of an acute thermal performance curve (TPC), where it increased until T_PEAK_, at which point *f*_Hmax_ declined with rising temperatures until the onset of cardiac arrhythmia (T_ARR_) (Fig. 6A). The TPC was more broadly shaped for wild fish, with a less apparent T_PEAK_ and more rapid onset of T_ARR_. There was evidence to support an effect of temperature on model selection for *f*_Hmax_, with a fourth-order polynomial curve determined to be the best-fit model by BIC (Supplementary Table S5). These models demonstrated that warm acclimation increased cardiac performance (i.e., *f*_Hmax_) and increased the upper thermal limits of the heart (i.e., T_PEAK_ and T_ARR_) (Fig. 6A–C). Peak *f*_Hmax_ ranged from 347.03 ± 23.05 bpm in fish taken directly from the wild (28 °C acclimatization) up to 415.50 ± 8.18 bpm in 33 °C acclimated fish, representing a 20% increase (Fig. 6, Table 2). Although upper thermal limits (T_PEAK_, T_ARR_) generally increased with warm acclimation, they only significantly differed between wild/28 °C acclimated and 33 °C acclimated for T_PEAK_ (ΔT = 2–2.7 °C) and between wild and 33 °C for T_ARR_ (ΔT = 1.7 °C) (Fig. 6B,C, Table 2). The thermal safety margin increased with acclimation temperature and was 28% greater in fish acclimated to 33 °C compared to wild-caught fish (P = 0.011; Fig. 6D).

**Table 2:** Summary statistics for acute cardiac thermal performance curves.

Metric	Wild (28 °C)	28 °C	29 °C	33 °C	Statistical parameters		
					df	F	P
N	7	8	10	10			
peak *f*_Hmax_ (bpm)	347.03 ± 23.05	382.23 ± 16.71	404.47 ± 9.11	415.50 ± 8.18	3	4.414	**0.011**
T_PEAK_ (°C)	34.83 ± 0.69	35.52 ± 0.32	36.47 ± 0.30	37.56 ± 0.21	3	9.961	** < 0.001**
T_ARR_ (°C)	36.34 ± 0.60	36.76 ± 0.31	37.34 ± 0.32	38.06 ± 0.21	3	4.364	**0.011**
TSM (31 °C)	5.34 ± 0.60	5.76 ± 0.31	6.34 ± 0.32	7.06 ± 0.21	3	4.364	**0.011**
TSM (33 °C)	3.34 ± 0.60	3.76 ± 0.31	4.34 ± 0.32	5.06 ± 0.21			

Metrics include sample size (N), peak maximum heart rate (peak *f*_Hmax_), the temperature of peak *f*_Hmax_ (T_PEAK_), the temperature at which arrhythmias began (T_ARR_), and thermal safety margin (TSM) as the difference between T_ARR_ and max environmental temperature (31 °C). Statistical results are from a one-way ANOVA.

Significant P-values are bolded.

## Discussion

This study provides evidence that hawkfish have a plastic response to simulated marine heatwave conditions. While temperature acclimation had a moderate impact on certain metabolic metrics (SMR, MMR, AAS, and FAS), it had a more pronounced effect on other important physiological processes. Recovery and digestion were found to be more costly even 1 °C above ambient conditions, and this appears to be exacerbated during the summer season. When testing the effect of relative meal size on digestion, we found that doubling the meal size did not increase digestion costs or SDA_peak_ at 33 °C. This study is also, to our knowledge, the first study to report data on coral reef fish cardiac performance during an acute temperature challenge, revealing that hawkfish have a plastic cardiac response to marine heatwave conditions, but this plasticity plateaus with increasing heatwave conditions. Overall, our study provides evidence that coral reef fish have impaired physiological performance under marine heatwave conditions, and experience important limitations with respect to digestion and recovery. These changes in physiology may impact their behavior, and could severely compromise their fitness and survival, altering their ecological roles within coral reef ecosystems during marine heatwaves.

Elevated temperatures are commonly known to impair the aerobic scope of coral reef fish^11,12,14,15^. However, this trend does not always hold true^32–34^ as observed here for arceye hawkfish. We found that the aerobic scope of the hawkfish was not significantly impaired at higher acclimation temperatures or across seasons, supporting the notion that aerobic scope may not always be the most informative performance metric across certain temperature ranges, or for certain species^35^. While examining a larger temperature range could show impaired aerobic scope in hawkfish at temperatures beyond those tested here, it may be more valuable to examine important aerobic activities within the context of aerobic scope at current and predicted future temperatures. In our study, hawkfish acclimated to warmer temperatures had less available scope for other activities during digestion and recovery, particularly during an extreme heatwave simulation (33 °C). This indicates that both recovery and digestion processes were more thermally sensitive to temperature changes than aerobic scope. Thus, the available scope for hawkfish to perform their ecological function as perching ambush predators on coral reefs is likely to be significantly reduced when digesting or recovering from exhaustive exercise at warming temperatures. Similar temperature-dependent effects on different performance metrics have been observed in other species with different ecological roles, including the swimming energetics of triggerfish and parrotfish, as well as the activity patterns of coral trout^36,37^.

As predators that perch on coral to hunt prey in exposed reef environments and engage in aggressive territorial interactions^38^, hawkfish rely on the capacity to rapidly swim and recover in a timely manner. Hawkfish acclimated to 29 °C took twice as long to recover to 75% AAS (the hypothesized threshold when hawkfish would be able to fully resume normal activities), and nearly six times as long at 33 °C compared to ambient winter conditions (27 °C). This indicates that fish exposed to more extreme marine heatwaves may have constrained energy to perform aerobic activities such as hunting and guarding territories (i.e., they have a reduced capacity to perform their ecological functions) and they remain vulnerable for an extended duration^39^. Somewhat surprisingly, fish in the summer acclimated to ambient conditions (28 °C) had impaired recovery compared to fish acclimated to the ambient winter conditions (27 °C). This could indicate seasonal effects on performance, with small differences in temperature having somewhat pronounced effects on recovery. Alternatively, this pattern could be related to differences in their thermal histories. The winter-tested fish (27 °C) had experienced a true heatwave, reaching 31 °C in the wild only a few months prior to collection, and may have been pre-acclimatized to warmer conditions (Fig. 1A).

Processing food increases metabolic rate because it requires the ingestion, digestion, absorption, and assimilation of nutrients^40–42^. Depending on the meal, conditions, and species, these costs can demand a vast scope, and this is especially true for predators^43^. Hawkfish that digested a meal of the same size (2% BM) at 33 °C compared to 27 °C had twice the metabolic cost during the peak of digestion (SDA_peak_), leaving 50% less aerobic scope available to them. The finding that higher temperatures induce greater metabolic rates during digestion aligns with the work done on other warm-adapted fishes, including lionfish (*Pterois* spp.)^43^, southern catfish (*Silurus meridionalis*)^44^, yellowfin tuna (*Thunnus albacares*)^45^, and the Caribbean neon goby (*Elacatinus lobeli*)^46^. Meal size (2 vs 4% BM) did not increase SDA_peak_ or SDA costs at 33 °C but ambient fish fed 4% BM had greater SDA_peak_ than those fed 2% BM. However, we did not test the same treatment (27 or 28 °C) across both 2 and 4% BM SDA trials, limiting our ability to assess if bigger meals disproportionately increase costs for fish acclimated to warmer temperatures. Additionally, the protein content was 37% higher in the scallop in 2022 compared to 2019, which would also increase SDA (Supplementary Table S1)^47^. Notably, the fish in our study were gavage-fed, which means they did not freely choose their relative meal size. However, the SDA_peak_/SMR remained ~ 2 across acclimation temperatures and relative meal sizes, indicating fish need to double their MO_2_ to digest a meal. Given the small factorial scope available to the fish during digestion (MMR/SMR–SDA_peak_/SMR) with the 4% BM ration size and at the warmer temperatures, hawkfish may choose smaller meals to retain scope for activity in the wild. This aligns with the finding that ectotherms eat less at supra-optimal temperatures^48^ potentially reducing their SDA_peak_ response to preserve aerobic scope for other activities^20^. If fish opt for smaller meals in higher temperatures to preserve energy for activity, their overall consumption decreases. This could have significant implications for their ecological role as predators and nutrient recyclers in the coral reef ecosystem^10,49^, which is particularly critical to corals during marine heatwaves^21^.

The ectotherm heart plays a crucial role in circulating blood containing oxygen, nutrients, wastes, hormones, and immune cells throughout the body. It is sensitive to temperature change and is considered the first organ system that fails under high temperatures, making it an ideal system for studying thermal tolerance^25^. Under acute warming, fish increase their heart rate to improve oxygen delivery to tissues^26,27^. In response to a prolonged thermal event, fish may undergo cardiac remodeling, which generally happens over the course of days to weeks^50^. In the case of a heatwave, which is prompt and temporary, the cardiac response is ideally rapid and reversible. By measuring the cardiac thermal limits of fish acclimated to different temperatures for one week, we captured the acclimation abilities of hawkfish to a relevant marine-heatwave timescale, where full thermal acclimation may not have yet occurred^51^.

The thermal tolerance limits of coral reef fish have been typically assessed in previous literature using measures such as CT_max_ or LT_50_, which have revealed limits from 34 to 44 °C depending on the species, life stage, or acclimation conditions^52–56^. While these metrics are informative in certain contexts^57^, they lack a direct link to the actual mechanisms driving thermal tolerance limits^58^, and occur at temperatures beyond which the heart has gone arrhythmic^59^, such that the fish is no longer functional. Instead, the cardiac thermal tolerance test may be more informative of functional thermal tolerance limits. Here, we showed that fish had a plastic response to warmer conditions by increasing their *f*_Hmax_, T_ARR_, and T_PEAK_, but they hit an upper thermal ceiling between 29 and 33 °C at which point cardiac performance was fixed. While the TSM for a 31 °C heatwave scenario ranged from 6 to 7 °C at the upper acclimation temperatures, hawkfish were increasingly unable to fully compensate. Though the CT_max_ of arceye hawkfish in Moorea has not been measured to our knowledge, the CT_max_ of the closely related whitespot hawkfish (*Paracirrhites hemistictus*), in Indonesia acclimated to 27.8 °C was 40.2 °C^53^, representing a 4 °C difference from the T_ARR_ of fish acclimated to 28 °C in our experiment. Even so, cardiac impairments (i.e., reduced scope for heart rate, diminished oxygen delivery) begin at temperatures below T_ARR_, making the functional thermal tolerance narrower than observed here^29^.

Of note is the finding that arceye hawkfish had some of the highest peak *f*_Hmax_ measured in fish thus far, reaching 415 bpm at 37.6 °C. In fact, their heart rates are greater than what would be expected given the peak maximum heart rates of other similar-sized fishes, including opaleye (*Girella nigricans*) acclimated to 20 °C: 205 bpm at 30 °C^59^, goldfish (*Carassius auratus*) acclimated to 28 °C: 210 bpm at 33.1 °C^60^, cyprinids (*Danio* spp.) acclimated to 28 °C: 256–323 bpm at 33–34 °C^61^, and killifish (*Fundulus heteroclitus*) acclimated to 33 °C: 244 bpm at 36.8 °C^62^. In South Africa, a sparid fish is already experiencing temperatures beyond its cardiac limits during a marine heatwave, threatening its survival and distribution^29^. Hawkfish, and likely other coral reef fish, are operating at a high and narrow temperature range where the heart nears its functional limits. The plasticity of the heart will determine whether these fish survive marine heatwaves and can function at such extremes, or if fish will need to move to cooler or deeper waters. While the plasticity of tropical fish hearts is understudied, research on salmonid (*Oncorhynchus* spp.) and zebrafish (*Danio* spp.) hearts indicates potential acclimation mechanisms ranging from changes at the molecular to the morphological level^25^. Warm acclimation changes cardiac mitochondrial metabolism and adrenergic sensitivity^25^. Over days to weeks, changes include a reduction in the relative ventricular mass, an increase in compact myocardium and capillary density within the compact myocardium, and changes in collagen fiber densities^50^. Although we do not examine the specific mechanism here, we show that arceye hawkfish have some capacity for cardiac thermal acclimation under heatwave conditions, but that plasticity reaches a ceiling beyond ~ 29 °C. Whether similar patterns exist in other coral reef species remains to be determined.

Conservation physiology informs us about how fish may respond to environmental and anthropogenic changes^63,64^. In addition to examining the acclimation response to marine heatwave conditions, we demonstrated the importance of assessing the cardiac thermal tolerance of non-acclimated fish captured directly from the wild. Despite having been acclimatized to a similar mean temperature of 28 °C, wild-caught fish had a lower peak *f*_Hmax_, T_ARR_, and T_PEAK_ than laboratory fish acclimated to 28 °C. This may be related to differences in thermal variation^65^, diet^51^, or other holding effects. Alternatively, fish may have had residual effects from the clove oil used to capture fish 3–8 h earlier in the day, though this is known to reduce T_PEAK_ and *f*_Hmax_ and not T_ARR_^28^.

A fish’s prior thermal experience may also impact its ability to perform during a marine heatwave. If fish are thermally acclimated for an extended period, fish may be able to fully compensate via physiological and morphological changes. The shorthorn sculpin, for example, fully thermally compensated certain performance metrics after eight weeks of acclimation to a warmer temperature^16^. In contrast, prolonged exposure to suboptimal temperatures could have deleterious impacts (e.g., impaired growth rates, gonad development, immune response, and swim performance)^12,66–68^. Unfortunately, we are unable to discern the 2019 marine heatwave effects from summer and winter conditions, and we are limited in our ability to directly compare performances between seasons due to differing temperature treatments. Even still, we found evidence for differences in metabolic performance between the two experimental timeframes. Seasonal variation in metabolism may be one possibility, where fish have reduced resting metabolic rates in the winter^69^. There is also the possibility that fish have elevated metabolism related to their spawning season^70^. Although the spawning season for arceye hawkfish remains unknown to us, the spawning season for long-nosed hawkfish (*Oxycirrhites typus,* Papua New Guinea) is in the summer^71^. On the other hand, the 2019 marine heatwave event could have had a legacy on fish performance, or fish that did not adapt relocated to deeper, cooler waters or died, and the most warm-adapted fish remained^72^. Thus, despite the small difference of approximately 1 °C in winter versus summer conditions, hawkfish may be more vulnerable to marine heatwaves in the summer when their metabolic demands are greatest, as they become more territorial and develop their gonads.

The plasticity of coral reef fish will largely determine how they fare in acute rapid environmental challenges posed by climate change. Although hawkfish showed some acclimation response, the hawkfish acclimated to 33 °C were compromised in activities that are fundamental to their role as predators including recovery from burst swimming and peak digestion. Finally, we found that cardiac plasticity plateaued at 29 °C. If hawkfish physiological performance is constrained during a marine heatwave in the wild, they may choose to adjust their diet type, diet quantity, or sacrifice energy toward other important activities. This would inherently alter how hawkfish interact with the reef and their important roles as invertivores and nutrient recyclers. The impact of such heatwaves on behavior and ecosystem function in the wild may have unanticipated consequences on the reef community, and these impacts may occur across fish species.

## Methods

### Site

This work took place at the University of California Gump Research Station on the volcanic high island of Moorea, French Polynesia. Concerning the spelling of Moorea, we followed the *Raapoto* transcription system that is adhered to by a large segment of the Tahitian community, but also recognize other community members follow the *Te Fare Vanā*'*a* transcription system where the island name is spelled with an ‘eta (i.e., Mo′orea) (see mcr.lternet.edu/spelling_of_Tahitian_place_names). Ocean temperature data (Fig. 1) were collected continuously (every 20 min) from 2005 to 2021 on a thermistor (SBE 39) mounted to the backreef of Moorea LTER2 (− 17.476993, − 149.802713) at 2 m depth as part of the Moorea Coral Reef Long-Term Ecological Research (LTER) time series^31^. One marine heatwave event associated with bleaching^73^ occurred Jan–Jul 2019 (Fig. 1A)^74^, and returned to average conditions approximately one week prior to the animal collections for Austral winter 2019 experiments (collection details below).

### Animal collection and husbandry

Arceye hawkfish (N = 75, 1–20 g) were collected on SCUBA or snorkel with clove oil (1:9, clove oil to 95% ethanol), hand nets, and slurp guns from the backreef (1–3 m depth) on the north shore of Moorea, French Polynesia (− 17.476477, − 149.818766) in two different seasons: the Austral winter (Jul–Aug 2019) and Austral summer (Nov–Dec 2022). Fish were transferred to a large cooler supplied with air stones (> 90% water air saturation) and transported back to the outdoor wet lab within 2 h. Following transport, fish were immediately transferred to shaded outdoor aquaria (500 l) supplied with ambient seawater pumped directly from the ocean and maintained at ambient water conditions for a minimum of 24 h and a maximum of two weeks before subjecting fish to their thermal acclimation treatments. Due to logistical constraints, holding time could not be tracked for individual fish and therefore could not be accounted for statistically, however, fish were randomly selected for acclimation to account for this. Fish in the wild consume a generalist diet of invertebrates and small fish^75^ but for consistency, were fed chopped scallops (*Argopecten purpuratus*) daily ad libitum. A subset of fish (N = 8) were captured one morning during Austral summer and underwent the cardiac thermal tolerance tests within 7 h of capture to represent Austral summer “wild” fish acclimatized to 28 °C (see below for details).

Fish were transferred into 65 l aquaria beneath an outdoor awning (4–6 fish per tank; 3 tanks per acclimation temperature), with three to four dead *Pocillopora* sp*.* coral heads included per tank to serve as shelter for these coral-associated fishes. Water temperature was either maintained at ambient temperature (winter: 27 °C, summer: 28 °C) or raised to the marine heatwave temperature (winter: 31 °C, summer: 29 or 33 °C) by 2°C per hour. These temperatures represent the current maximum and projected climate change (summer: + 1, + 5 °C; winter: + 4 °C) temperatures for this population during the different seasons^76^. Tanks were maintained above 80% of oxygen saturation and subject to the natural photoperiod (13 h light:11 h dark). Given that a marine heatwave may last from 5 days to several weeks^3^, fish were acclimated to their temperature treatment for 1 week before respirometry trials^14^. Fish acclimated to 33 °C were fed twice daily to ensure they had the same access to food relative to their metabolism as fishes at lower temperatures. All experimental protocols were approved by the Institutional Animal Care and Use Committee at the University of California, Santa Barbara (protocol #955-955.1) and are in accordance with relevant guidelines (including ARRIVE guidelines) and regulations.

### Intermittent flow respirometry

Oxygen consumption rates (MO_2_) were measured in a custom-made intermittent flow respirometry system to measure maximum metabolic rate (MMR), recovery, standard metabolic rate (SMR), and specific dynamic action (SDA) in individual fish. A header tank maintaining ambient (27–28 °C) or heated water (29, 31, or 33 °C) in an open circuit supplied 102 l tubs or a water table containing submerged respirometers custom-made from polyvinyl tupperware (Lock & Lock; Seoul, South Korea). Respirometers (13 × 8.7 × 5.5 cm, 566 total l; or 13.4 × 9 × 5.8 cm, 690 total l) were plumbed with PVC tubing to recirculation pumps (Eheim Universal 300 pump, flow rates averaged 2 l min^−1^) and flush pumps (Eheim Universal 600 pump, flow rate to each respirometer averaged 1.1 l min^−1^). Because of the relatively small size of *P. arcatus,* flush pumps were divided between two to four respirometers. FireSting robust Oxygen probes (PyroScience, Aachen, Germany) were fitted into the recirculation loop and measured oxygen levels continuously. Recirculation pumps continuously pumped water throughout a closed loop, and flush pumps were set on a timer to automatically turn on intermittently to flush fresh seawater into the respirometers and ensure dissolved oxygen did not reach below 70%. Shade cloth covered the respirometers to prevent disturbance and excess light exposure.

To account for bacterial respiration, background was measured in all respirometers for a minimum of three cycles before and after each full set of respirometry trials (before MMR and after SDA). Additionally, the entire setup was drained, rinsed, and cleaned with freshwater and bleach between each trial of 8–12 fish to minimize bacterial growth.

Fish were fasted 24 h before respirometry to assume a post-absorptive state^77^. This was an assumption made prior to having estimated SDA duration in hawkfish and was maintained for standardization. Trials began between 11:00 and 16:00. Maximum metabolic rate (MMR) was measured at the beginning of the respirometry trial, except for two trials (N = 4 fish (27 °C), N = 4 fish (31 °C)) where MMR measurements followed SMR measurements due to logistical constraints, but this timing did not affect MMR measurements at either temperature (T-tests, P = 0.631 (27 °C), P = 0.621 (31 °C)). MMR was induced by hand-chasing an individual fish in a bucket for 3 min followed by 1 min of air exposure in a hand net and immediately placing the individual in a respirometer^78^. MO_2_ was measured for 5–7 min to measure MMR and then chambers were flushed with fresh seawater. Following MMR measurements for each fish, the flush pumps were automated to reoxygenate the respirometers with fresh seawater in 15 min intervals of flush:measure cycles (e.g., 5 min flush: 10 min measurement; 6 min flush:9 min measurement). Fish were kept in respirometers overnight for 18–20 h for recovery and SMR measurements, with flush cycles modified as needed to maintain oxygen saturation above 70%.

Digestion is a metabolically expensive process that involves the breakdown, transport, and synthesis of food molecules^40^. The oxygen cost of digestion and assimilation is termed the specific dynamic action (SDA). Fish would not freely feed in their respirometers or in isolation in a tank, so they were gavage-fed for SDA measurements, which is a common approach for delivering food to fishes for SDA experiments^79^. After the overnight recovery measurements, each fish was removed from the respirometer, anesthetized in clove oil (2019: 20 mg l^−1^, 2022: 10 mg l^−1^, 1:9 clove oil to 95% ethanol), weighed, and gavage fed 2 or 4% of their body weight in scallops with forceps. These different relative meal sizes were chosen to mimic a relatively small and large meal and are typical in digestion studies^41^. Food loss was monitored and estimated in an aerated recovery bucket for 10 min before returning the fish to the respirometer. Any regurgitated food was weighed and re-fed to the individual, which was typically necessary a second time for about 50% of the fish and a third time for 5% of the fish. Fish were again monitored for 10 min before being returned to their respective respirometer. MO_2_ measurement cycles began after all fish per trial were fed, which occurred within a 2-h time frame. MO_2_ was measured for 40–44 h to estimate SDA (flush:measure cycles as described above for recovery and SMR).

Following SDA, fish were either euthanized (immersed in MS-222, 500 mg l^−1^) and dissected (N = 32), sham-fed (N = 8), or underwent the cardiac thermal tolerance test (N = 30). Fish were sham fed to determine the duration of the stress and handling response induced by the anesthesia (2019: 20 mg l^−1^, 2022: 10 mg l^−1^, 1:9 clove oil to 95% ethanol) and gavage procedure during which forceps were used to open their mouths to mimic gavage feeding. Fish were recovered in an aerated bath, a subset (N = 2) underwent the gavage procedure a second time and then fish were returned to respirometers to measure MO_2_ for 5–18 h (Supplementary Fig. S2). To verify that fish recovered from clove oil within 5 h during the first year of trials, the longer time frame was selected for the following year.

### MO_2_ analysis

MO_2_ data were analyzed and visualized in R (version 4.2.1) using the package *AnalyzeResp*^80^. Mass-specific MO_2_ (units: mg O_2_ kg^−1^ min^−1^) was calculated from the change in concentration of O_2_ over time (∆O_2_) in the respirometer using MO_2_ = (∆O_2_ × (v_R_ − v_F_))/m, where v_R_ is the respirometer volume, v_F_ is the volume of the fish (L, assuming 1 kg = 1 l), and m is the fish mass (kg). All measurement period dissolved oxygen regressions were visually assessed to ensure each O_2_ slope was linear and negative. All MO_2_ values were corrected for microbial background respiration. Background respiration was calculated based on a first-order exponential curve calculated between the average initial and end background measurements for each set of respirometers and then subtracted from the slope of each associated MO_2_ measurement. At the onset of trials, background respiration levels typically accounted for 10% of the observed respiration rates for the fish and grew to as high as 60% by the trial’s conclusion. MO_2_ values were assessed for body mass effects on oxygen consumption rates (SMR and MMR). Using the Bayesian Information Criterion (BIC), we determined that SMR values were isometric, whereas MMR values scaled allometrically and required a scaling correction (Supplementary Fig. S1). The two best-fit models (ΔBIC < 7) used the hawkfish data (scaling coefficient = 0.69) or the universal scaling coefficient for fish (scaling coefficient = 0.89)^81^. Due to the small range in body mass, we opted for the universal metabolic scaling coefficient of 0.89 for MMR and used 5 g as the common body mass. The corrected MMR was used in all subsequent calculations and statistics.

MMR was selected as the first MO_2_ measurement post-exhaustive exercise. This was the highest MO_2_ value for all fish except for 11% of fish (N = 6) which experienced the highest MO_2_ post-feeding during the 3 h recovery from anesthesia and handling. For these individuals, the first MO_2_ post-exercise was still selected as ‘MMR’. MMR was calculated using a sliding window analysis (≥ 120 s), where each sliding window began at the start of the measurement period and moved in 1 s increments across the measurement cycle, selecting the steepest ∆O_2_ with an R^2^ > 0.9 as MMR^82,83^. Whether this exhaustive exercise elicited true MMR is unclear, but manual chasing to exhaustion provides the most reliable measure of MMR in species that do not swim for prolonged periods^78^, such as hawkfish. SMR was calculated as the lowest 10% quantile of all validated MO_2_ measurements post-exhaustive exercise and post-feeding with R^2^ > 0.95. Absolute aerobic scope (AAS) values were calculated as MMR–SMR for each individual, and factorial aerobic scope (FAS) as MMR/SMR. AAS represents the aerobic capacity of the fish to perform activities beyond standard (e.g., growth, swimming, digestion)^27,35^. FAS represents the aerobic capacity of the fish relative to its standard rate of oxygen uptake^82^.

Recovery from exercise is a metabolically expensive process that restores homeostasis by clearing lactate and restoring oxygen stores, glycogen, high-energy phosphates, and osmoregulatory balance^84^. During recovery, fish are vulnerable and may forgo important activities (e.g., forage, compete for territory, find mates)^39^. Hawkfish are ambush predators and therefore, immediate recovery may be a more relevant metric and was estimated rather than the total excess post oxygen recovery (EPOC)^35,85^. Short-term recovery was calculated in three ways: (1) MO_2_ over time, by fitting individual biexponential curves to each acclimation temperature as determined by the BIC. (2) Time to FAS = 2, as MMR/MO_2_ for each individual during the recovery following exercise and pooled in 10–15 min time blocks for each treatment. Time to reach FAS = 2 was selected as a recovery threshold because this is the point at which fish can fully digest a meal and likely resume foraging^86–88^. (3) %AAS, calculated as (MMR-MO_2_)/AAS at each MO_2_ measurement for each fish. Logarithmic growth curves were fitted to the recovery data using the nls function from the *stats* package (R Core Team, 2022). The threshold of 75% AAS was selected as a standardized metric under the assumption that hawkfish would resume normal activity (e.g., hunt, compete for territories, swim rapidly) between 50 and 90% AAS^89^. How hawkfish or species with similar life histories prioritize metabolic demands at supra-optimal temperatures is unknown, but based on work with more active species, this threshold is a moderate starting point to compare recovery across temperatures^9^.

Based on the sham feeding trials, clove oil and handling had a 2–3 h effect on MO_2_ (Supplementary Fig. S2). Therefore, MO_2_ measurements included in the SDA analyses for each fish began 3 h after feeding. SDA_peak_ was calculated as the maximum postprandial MO_2_, and the associated time from feeding to reach peak SDA was termed ‘time-to-SDA_peak_’. The duration of SDA (SDA_dur_) was calculated as the number of hours between time fed and the first point of MO_2_ to reach the lowest 10% quantile of recorded postprandial MO_2_ values (SMR_SDA_). SMR_SDA_ was statistically the same as SMR calculated as described above (t-test, P = 0.554). SDA was calculated by integrating the area beneath postprandial MO_2_ minus SMR_SDA_ and began at the time of feeding with a line extrapolated from SMR to the first analyzed MO_2_ measurement (i.e., 3 h) (Fig. 2). The remaining scope for activity during SDA_peak_ indicates the percentage of energy fish have available to them during the most metabolically expensive part of digestion. This was calculated as (mean SDA_peak_ − mean SMR)/(mean AAS) × 100 for each treatment (temperature × relative meal size). SDA_peak_/SMR indicates the proportion of energy allocated during the most metabolically expensive part of digestion. An SDA_peak_/SMR = 2 suggests fish need to double their MO_2_ to digest a meal. The cost of SDA (SDA_coeff_) represents the percentage of energy consumed and was calculated as SDA_coeff_ = (E_SDA_/E_meal_) × 100, where E_SDA_ is the energy spent on SDA, assuming 1 g of O_2_ is associated with the release of 13.6 kJ of energy^90^, and E_meal_ is the energy of the scallop meal, calculated as the mass of scallop fed multiplied by its average gross energy density (3.87 kJ g^−1^) and a 0.8 correction factor to account for indigestible energy^42^.

### Cardiac thermal tolerance test

Cardiac function governs whole-organism performance and its response to a controlled acute temperature increase (i.e., the cardiac thermal tolerance test) reveals the acclimation potential of populations to respond to climate change scenarios^86^. In summer 2022, a subset of fish from respirometry (N = 30) and from the wild (N = 8) underwent the cardiac thermal tolerance test^28,59,91^. Since fish were transferred from the respirometer to the cardiac thermal tolerance test, we could ensure fish were starved for 40–44 h. However, for the ‘wild’ treatment, we could only verify fish hadn’t eaten since the morning of collection (2–7 h). Fish were individually removed from a respirometer or the “wild” holding tank, anesthetized in seawater containing 80 mg l^−1^ MS-222 buffered with NaHCO_3_, and then placed ventral side up in a sling in a water bath (10 l seawater containing buffered 65 mg l^−1^ MS-222). Water was circulated past the gills for constant irrigation, and an airstone maintained oxygenation. Stainless steel needle tip electrodes (ADInstruments Inc., Colorado Springs, CO, USA) were inserted beneath the skin to detect an ECG signal that was amplified with a Dual Bio Amp amplifier (ADInstruments Inc.) and filtered (filters: 60 Hz Notch filter; mains filter; low pass: 2 kHz; high pass: 10 Hz; range: 2 mV)^59,65^.

After a 15 min equilibration period at the acclimation temperature, atropine sulfate was injected intraperitoneally (1.2 mg kg^−1^ in 0.9% NaCl) to block vagal tone, and 15 min later, isoproterenol was injected intraperitoneally (4 μg kg^−1^ in 0.9% NaCl) to maximally stimulate β-adrenoreceptors. These drug concentrations were tested prior to the experiment to ensure a double dose did not further increase heart rate (*f*_H_; beats min^−1^). After another 15 min, the test began, and water was heated by 1 °C every 6 min by running recirculating heated water through a stainless-steel coil in each water bath. At each 1 °C interval, *f*_Hmax_ and temperature were stabilized and recorded. The test ended after the onset of cardiac arrhythmia (T_ARR_), as indicated by a transition from rhythmic to arrhythmic beating or a missed QRS peak and precipitous decline in heart rate for all tested fish^28^. Individuals were then euthanized (immersed in MS-222, 500 mg l^−1^), measured for total length, and dissected to verify their digestion status.

### Cardiac thermal tolerance test analysis

ECG analyses were performed in LabChart Software (AD Instruments, Dunedin, New Zealand). *f*_Hmax_ was calculated for each temperature increment from a continuous 15 s measurement. Due to the relatively high acclimation temperatures and limited data points, the Arrhenius breakpoint temperature was not determined. Peak maximum heart rate (*f*_Hmax_) was determined from the highest *f*_Hmax_ recorded over a 15 s measurement, and peak temperature (T_PEAK_) was defined as the temperature corresponding to peak *f*_Hmax_. T_ARR_ was determined as the temperature when the heartbeat transitioned from rhythmic to arrhythmic beating, when the trace missed a QRS peak, or when there was a precipitous decline in *f*_Hmax_^28^. Of the 38 tests, 3 were deemed unusable due to poor ECG conductivity. Thermal safety margin (TSM) was calculated as T_ARR_ − max environmental temperature. Polynomial curves were fitted to *f*_Hmax_ data and compared using BIC, where the fit with the lowest BIC score was assigned the best-fit model.

### Statistical analyses

Data were analyzed using R version 4.2.1. Values are presented as mean ± standard error of mean (SEM), and statistical significance was set at P < 0.05. Metrics were investigated for normality using Shapiro–Wilk tests and quantile–quantile plots, and for heteroscedasticity using Levene’s test.

To examine how whole-animal physiological performance varied across acclimation temperatures, we used a one-way ANOVA to compare SMR, MMR, AAS, and FAS separately across seasons. Fish fed 2% BM were also assessed for differences in SDA, SDA_peak_, SDA_dur_, time-to-SDA_peak_, and SDA_coeff_ between acclimation temperatures each season using independent t-tests. To determine if relative meal size (2 vs 4% BM) increased costs disproportionately, SDA, SDA_peak_, SDA_dur_, time-to-SDA_peak_, and SDA_coeff_ values were compared across acclimation temperatures and relative meal sizes using a two-way ANOVA. Finally, to assess the difference in upper thermal tolerance cardiac limits across acclimation temperatures, a one-way ANOVA was used to compare *f*_Hmax_, T_PEAK_, and T_ARR_ across acclimation temperatures. All ANOVAs were followed by post-hoc Tukey HSD. For nonsignificant interactions, the interaction was removed from the model. When data are collected from two separate field seasons (Austral winter and summer), data are presented separately by season.

### Supplementary Information

Supplementary Information.

## Data Availability

The data that support the findings of this study will be openly available in Dryad at 10.5061/dryad.wdbrv15vm upon acceptance. The temporary reviewer link is: https://datadryad.org/stash/share/pYJSneZ6hTNbXFb_-GvmrmL8Tyz0vPt5N11InLVKhfg.
