# Supplementary material for: Despite plasticity, heatwaves are costly for a coral reef fish
Source: Sci Rep. 2024 Jun 10;14:13320. doi: 10.1038/s41598-024-63273-8 (PMC11164959; doi:10.1038/s41598-024-63273-8)
Supplement: Supplementary file 1 — Supplementary Information. [file 41598_2024_63273_MOESM1_ESM.pdf]

Van Wert et al. 2024 Supplementary Material

Despite plasticity, heatwaves are costly for a coral reef fish

Jacey C. Van Wert, Kim Birnie-Gauvin, Jordan Gallagher, Emily A. Hardison, Kaitlyn Landfield, Deron E. Burkepile, Erika J. Eliason

Table S1: Proximate analysis of scallop (*Argopecten purpuratus*) from 2019 (winter) and 2022 (summer). Analyses followed methods outlined in Van Wert et al. (2023).

Sample size	Year	Protein (% DW)	Lipid (%DW)	Carbohydrate (% DW)
3	2019	25.85 ± 2.52	NA	4.05 ± 1.33
5	2022	35.29 ± 2.39	4.51 ± 0.40	NA

All values but water (% total) are presented in % dry weight (DW) and are represented as the mean ± SEM of each replicate.

Table S2: Summary statistics for metabolism.

Metric	27°C (winter)	28°C (summer)	29°C (summer)	31°C (winter)	33°C (summer)	Statistical parameters	df	F	P
MMR (mg O_2 kg^{-1} min^{-1})	13.89 ± 1.02 (13)	11.04 ± 0.78 (12)	12.72 ± 0.94 (9)	14.88 ± 1.22 (10)	13.89 ± 0.62 (20)	S	2	4.01	**0.026**
						W	1	0.68	0.422
SMR (mg O_2 kg^{-1} min^{-1})	2.21 ± 0.26 (10)	2.91 ± 0.23 (12)	3.36 ± 0.09 (9)	3.38 ± 0.48 (9)	4.66 ± 0.29 (20)	S	2	12.66	**< 0.001**
						W	1	2.82	0.114
AAS (mg O_2 kg^{-1} min^{-1})	11.53 ± 1.31 (10)	8.14 ± 0.76 (12)	9.36 ± 0.9 (9)	12.03 ± 1.61 (7)	9.23 ± 0.58 (20)	S	2	0.79	0.461
						W	1	0.06	0.809
FAS	6.65 ± 0.73 (10)	4.08 ± 0.5 (12)	3.78 ± 0.25 (9)	5.72 ± 1.17 (7)	3.31 ± 0.41 (20)	S	2	0.879	0.423
						W	1	0.52	0.484

Represented are mean ± SEM with sample size in parentheses for each acclimation temperature and one-way ANOVA results across seasons are presented. SMR = standard metabolic rate; MMR = maximum metabolic rate; AAS = absolute aerobic scope; FAS = factorial aerobic scope; df = degrees of freedom, F = F-value, P = P-value. 'S' indicates statistics calculated for summer acclimated fish (28, 29, 33°C) and 'W' indicates statistics calculated for winter acclimated fish (27, 31°C).

Table S3: Summary statistics for SDA at 2% BM.

Metric	27°C (winter)	29°C (summer)	31°C (winter)	33°C (summer)	Statistical parameters			
	2% BM				Season	df	T	P
n	10	9	8	7				
SDA (mg O_2 kg^{-1})	31.23 ± 4.24	48.58 ± 6.96	30.3 ± 4.78	36.62 ± 12.77	S<br>W	14<br>16	0.40<br>0.15	0.397<br>0.886
SDA_{dur} (h)	28.1 ± 1.42	31.94 ± 1.44	24.12 ± 2.38	25.83 ± 6.99	S<br>W	13<br>16	1.04<br>1.50	0.317<br>0.153
SDA_{peak} (mg O_2 kg^{-1} min^{-1})	4.95 ± 0.47	8.63 ± 0.78	7.26 ± 0.46	9.35 ± 1.13	S<br>W	14<br>16	-0.54<br>-3.46	0.598<br>**0.003**
time-to-SDA_{peak} (h)	13.6 ± 2.94	6.61 ± 2.9	7.94 ± 1.73	3.5 ± 0.94	S<br>W	14<br>16	0.91<br>1.55	0.377<br>0.140
SDA_{coeff} (%)	0.69 ± 0.09	1.07 ± 0.15	0.67 ± 0.11	0.8 ± 0.28	S<br>W	14<br>16	0.87<br>-0.15	0.397<br>0.886

Represented are mean ± SEM and summary statistics from independent t-tests for each parameter compared between acclimation temperatures within each season. SDA = specific dynamic action, SDA_{dur} = duration of SDA, SDA_{peak} = peak MO_2 during SDA, time-to-SDA_{peak} is hours to peak SDA, SDA_{coeff} = coefficient of specific dynamic action, df = degrees of freedom, T = T-value, P = P-value. Significant values are bolded. 'S' indicates statistics calculated for summer acclimated fish (29, 33°C) and 'W' indicates statistics calculated for winter acclimated fish (27, 31°C).

Table S4: Summary statistics for SDA metabolism at 2 vs. 4% BM.

Metric	27°C (winter)	28°C (summer)	33°C (summer)		T-test results for 33°C		
	2% BM	4% BM	2% BM	4% BM			
n	10	10	7	11	t	df	P
SDA (mg O_2 kg^{-1})	31.23 ± 4.24	57.44 ± 8.43	36.62 ± 12.77	52.56 ± 6.28	-1.20	8.94	0.292
SDA_{dur} (h)	28.1 ± 1.42	33.28 ± 1.01	25.83 ± 6.99	25.94 ± 1.46	-0.02	13	0.985
SDA_{peak} (mg O_2 kg^{-1} min^{-1})	4.95 ± 0.47	7.16 ± 0.61	9.35 ± 1.13	9.98 ± 0.92	-0.45	16	0.660
time-to-SDA_{peak} (h)	13.6 ± 2.94	6.15 ± 1.6	3.5 ± 0.94	5.23 ± 1.52	-0.84	16	0.416
SDA_{coeff} (%)	0.69 ± 0.09	1.26 ± 0.19	0.8 ± 0.28	1.15 ± 0.14	-1.25	16	0.230

Represented are mean ± SEM. For 33°C are also summary statistics from t-tests for each parameter. SDA = specific dynamic action, SDA_{dur} = duration of SDA, SDA_{peak} = peak MO_2 during SDA, time-to-SDA_{peak} is hours to peak SDA, SDA_{coeff} = coefficient of specific dynamic action, df = degrees of freedom, F = F-value, P = P-value. Significant values are bolded.

Table S5: BIC outputs for polynomial cardiac thermal tolerance curves.

Model	Formula	df	BIC	ΔBIC
Model 1	bpm~poly(acute_temp, 4) * treatment, random = ~1|fish_id	22	3377.44	0
Model 2	bpm~poly(acute_temp, 3) * treatment, random = ~1|fish_id	18	3404.02	26.59
Model 3	bpm~poly(acute_temp, 2) * treatment, random = ~1|fish_id	14	3468.16	90.73
Model 4	bpm~poly(acute_temp, 4) + treatment, random = ~1|fish_id	10	3485.47	108.04
Model 5	bpm~poly(acute_temp, 3) + treatment, random = ~1|fish_i	9	3496.49	119.05
Model 6	bpm~poly(acute_temp, 2) + treatment, random = ~1|fish_id	8	3547.55	170.11
Model 7	bpm~ acute_temp * treatment, random = ~1|fish_id	10	3711.99	334.55
Model 8	bpm~ acute_temp + treatment, random = ~1|fish_id	7	3715.46	338.02

Represented are model formulas and BIC model selection. df = degrees of freedom, BIC = Bayesian Information Criterion, ΔBIC = BIC(model)—BIC(min BIC value), bpm = maximum heart rate in beats per min, acute_temp = acute temperature, treatment indicates acclimation (wild, 28, 29, or 33°C), fish_id = individual fish.

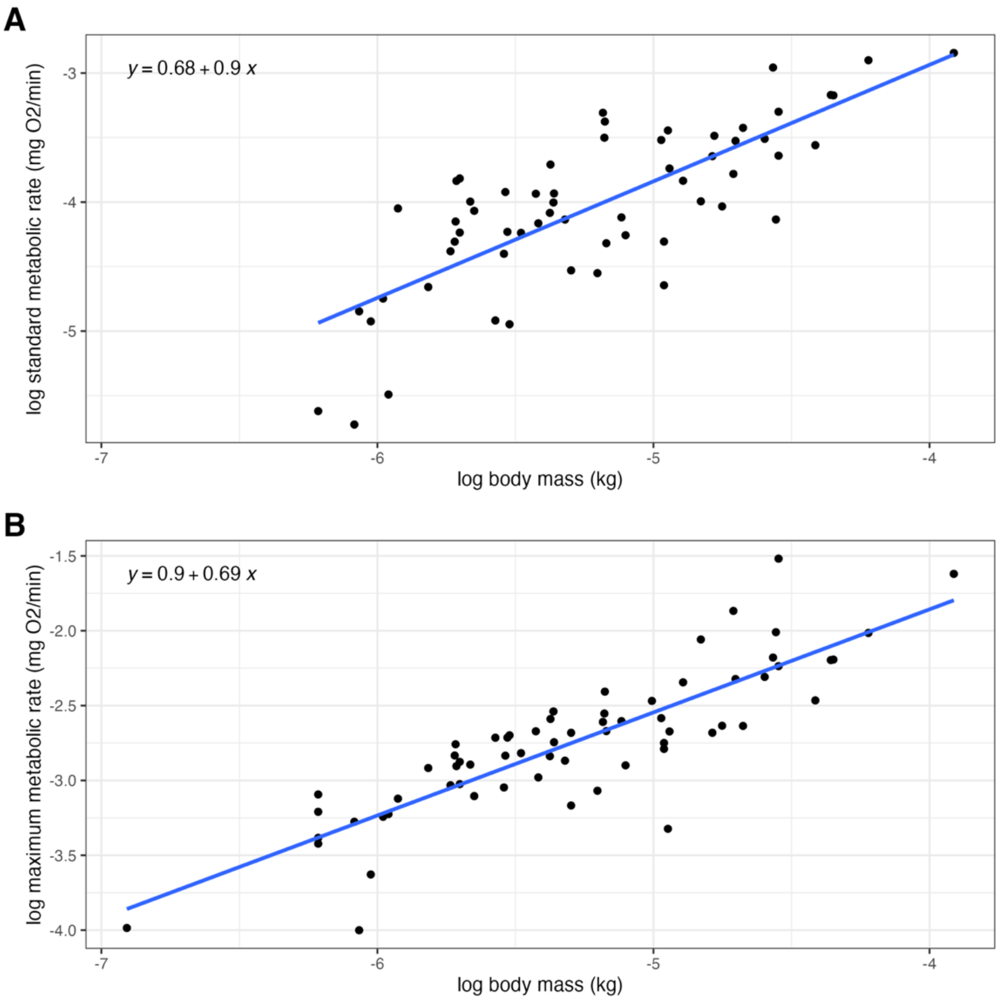

Figure S1. Log-log plots of log mass-specific standard metabolic rate (top) and mass-specific maximum metabolic rate (bottom) vs log body mass (kg) with fitted linear regression and associated equation.

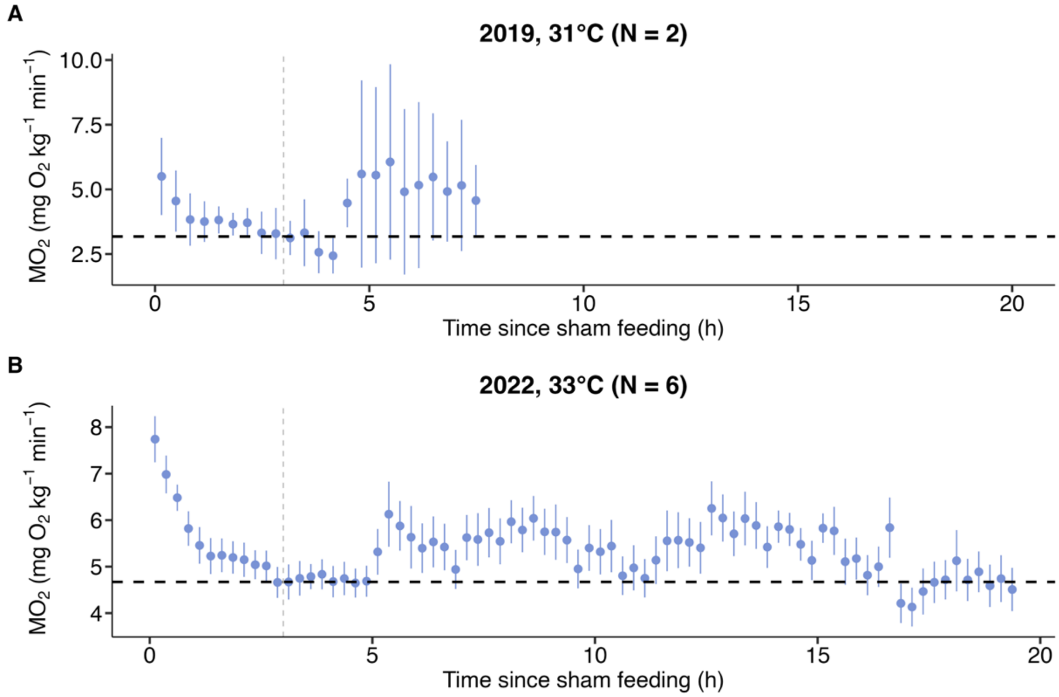

Figure S2. MO_2 after sham feeding to determine the effect of the clove oil anesthetic and gavage handling. Mean ± SEM MO_2 of (A) 2 individuals acclimated and tested at 31°C in winter 2019 for 6 h and (B) 6 individuals acclimated and tested at 33°C in summer 2022 for 18 h. The horizontal line indicates the average SMR for each treatment and the vertical line denotes the 3 h timepoint, where the anesthesia, gavage and handling effects are deemed negligible.
